# Association of Obstructive Sleep Apnea With Cardiovascular Events in Acute Coronary Syndrome Patients With or Without Excessive Daytime Sleepiness: A Prospective Cohort Study

**DOI:** 10.31083/RCM33439

**Published:** 2025-07-28

**Authors:** Yuyao Qiu, Zexuan Li, Wen Hao, Xiaochen Liu, Qian Guo, Yingying Guo, Bin Que, Wei Gong, Wen Zheng, Xiao Wang, Shaoping Nie

**Affiliations:** ^1^Center for Coronary Artery Disease, Division of Cardiology, Beijing Anzhen Hospital, Capital Medical University, 100029 Beijing, China; ^2^Department of Cardiology, Beijing Hospital, National Center of Gerontology, Institute of Geriatric Medicine, Chinese Academy of Medical Sciences, 100730 Beijing, China; ^3^Cardiometabolic Medicine Center, Fuwai Hospital, National Center for Cardiovascular Diseases, Chinese Academy of Medical Sciences and Peking Union Medical College, 100037 Beijing, China

**Keywords:** obstructive sleep apnea, acute coronary syndrome, excessive daytime sleepiness, cardiovascular events

## Abstract

**Background:**

Excessive daytime sleepiness (EDS) is a commonly observed symptom in people with obstructive sleep apnea (OSA). However, the impact of EDS on the outcome of patients with acute coronary syndrome (ACS) and OSA is not known. Therefore, this study aimed to investigate the association between OSA and cardiovascular events in ACS patients with or without EDS.

**Methods:**

This cohort study prospectively enrolled eligible ACS patients who underwent cardiorespiratory polygraphy during hospitalization between June 2015 and January 2020. We defined OSA as an apnea–hypopnea index (AHI) ≥15 events per h. EDS was described as having an Epworth Sleepiness Scale score ≥10. Major adverse cerebrovascular and cardiovascular events (MACCEs) were the primary outcome and included cardiovascular death, stroke, myocardial infarction, ischemia-driven revascularization, or hospitalization for heart failure or unstable angina.

**Results:**

The final study cohort comprised 1154 participants, of whom 398 (34.5%) had EDS, and 607 (52.6%) had OSA. During the median follow-up period of 2.9 years (interquartile range 1.5, 3.6), OSA was associated with a significantly increased risk of MACCEs in patients without EDS (adjusted hazard ratio (HR) = 1.42, 95% CI: 1.01–2.02, *p *= 0.046), but not in patients with EDS (adjusted hazard ratio HR = 1.05, 95% CI: 0.67–1.66, *p *= 0.84).

**Conclusions:**

OSA was associated with an elevated risk of MACCEs In ACS patients without EDS but not those with EDS. Therefore, screening for OSA should be performed in ACS patients without EDS, and future trials should prioritize such high-risk patients.

**Clinical Trial Registration:**

NCT03362385, https://clinicaltrials.gov/study/NCT03362385.

## 1. Introduction 

Obstructive sleep apnea (OSA) is a prevalent sleep disorder that is characterized by repeat occurrences of partial or complete obstruction of the upper airway during sleep. Recent studies have highlighted the substantial public health cost associated with OSA, with the global prevalence in 30–69 year old adults estimated to be 940 million [1]. The incidence of OSA in patients with acute coronary syndrome (ACS) was found to be 36–63% in different races [2]. Several different studies have reported that OSA has prognostic significance in ACS patients [3, 4]. However, there may be specific subsets (e.g., with different symptoms or comorbidities) that are more likely to be affected by OSA [5, 6, 7] and have yet to be identified.

Patients with OSA are commonly accompanied by sleep disruption, which frequently results in excessive daytime sleepiness (EDS). EDS may adversely impact daily performance, emotional states, and various facets of overall well-being [8, 9, 10]. Neuroimaging also reveals changes in the white matter and gray matter within the cerebral structure of individuals presenting with both OSA and EDS. A higher mean diffusivity and significant gray matter concentration deficits were detected in the EDS group by whole-brain analysis [11, 12]. Recent work has also proposed an association between EDS and major adverse cerebrovascular and cardiovascular events (MACCEs) after myocardial infarction (MI) [13]. Currently, therapy with continuous positive airway pressure (CPAP) or pharmacological treatments are usually offered to OSA patients with EDS. However, it is still unclear if the effect of OSA on cardiovascular outcome differs according to the EDS status. In the present study we therefore investigated whether OSA was associated with cardiovascular events in ACS patients who did or did not have EDS. To achieve this, we conducted post-hoc analyses of an earlier OSA-ACS study.

## 2. Materials and Methods

### 2.1 Study Design and Participants

The OSA-ACS project (NCT03362385) was a large, prospective cohort study that that has been reported previously [5]. The study enrolled eligible ACS patients aged 18 to 85 years at the Beijing Anzhen Hospital, Capital Medical University with the aim of investigating possible associations between OSA and cardiovascular outcomes. Data collection occurred between June 2015 to January 2020, with the participants followed until December 2020. The exclusion criteria were: individuals with cardiogenic shock, cardiac arrest, cancer, patients with unsuccessful sleep monitoring due to unsatisfactory or inadequate signal recording, and patients who received regular CPAP treatment. Also excluded were patients who had predominantly central sleep apnea (≥50% central events and a central apnea-hypopnea index (AHI) ≥10 events per hour), as well as patients lost to follow-up following hospital discharge.

MI diagnosis followed the universal definition of myocardial infarction, requiring both clinical evidence of acute myocardial ischemia and cardiac biomarker changes [14, 15]. Unstable angina (UA) diagnosis was based on the guideline, primarily emphasizing ischemic symptoms without mandatory biomarker elevation [16].

This investigation followed the principles of the Declaration of Helsinki, and was approved by the Ethics Committee, Beijing Anzhen Hospital, Capital Medical University (2013025). Written informed consent was obtained from all participants.

### 2.2 Sleep Study and Procedures

Following clinical stabilization in the hospital, all eligible participants underwent an overnight sleep study with a Type III portable cardiorespiratory polygraphy instrument (ApneaLink Air; ResMed, San Diego, California, USA). The mean time interval from admission to sleep study was 2 ± 1 days. This was independently applied at bedtime by trained research personnel. Data output from this device was collected by researchers who were blinded to patient information. Recorded parameters included nasal airflow, snoring episodes, heart rate, thoraco-abdominal movement, and nocturnal oxygen saturation (SaO2). Apnea was defined as airflow cessation lasting >10 seconds (obstructive in the presence of thoraco-abdominal movement, and central in the absence of thoraco-abdominal movement). Hypopnea was defined as a >30% decrease in airflow for >10 seconds, with a >4% reduction in arterial SaO2. We defined AHI as the number of apnea and hypopnea events per hour recorded over the total period. As per previous guidelines, we classified OSA as an AHI ≥15 events/h. Conversely, individuals that had an AHI <15 events/h were classified non-OSA [3, 17, 18, 19]. Standard treatment was given to participants during hospitalization for the ACS event, as per current guidelines [20, 21].

### 2.3 Epworth Sleepiness Scale

Sleepiness was self-reported using the Epworth Sleepiness Scale (ESS). Patients subjectively assessed their daytime sleepiness by completing the ESS questionnaire [22]. This scale includes eight questions, each relating to the risk of falling asleep in different situations over the past month. A categorization of EDS was assigned to patients who scored at least 10 out of a possible total of 24.

### 2.4 Follow-up and Endpoints

All eligible patients were monitored until December 2020. Follow-up assessments were performed 1, 3 and 6 months post-discharge, then subsequently each year. Adverse clinical events were registered through clinic visits, review of medical records, and phone interviews by researchers who were blinded to sleep data for each patient. The primary endpoint (MACCEs) included cardiovascular death, stroke, ischemia-driven revascularization, MI, and hospitalization due to UA or heart failure (HF). As detailed previously [23], secondary endpoints were single components of the primary endpoint, all-cause death, all repeat revascularization, and an amalgam of all events. Endpoints were established using definitions outlined by the Standardized Data Collection for Cardiovascular Trials Initiative [24]. Only the first occurrence from baseline was counted when the patient experienced multiple events. Events and source documents were assessed independently by researchers who were blind to the sleep study results.

### 2.5 Statistical Analyses

Quantitative data was presented as mean ± SD, or median (Q1, Q3), and evaluated by Student’s *t*-test or Mann-Whitney U test. Qualitative data was presented as a percentage and analyzed by Chi-square test or Fisher’s exact test. Kaplan-Meier analysis was performed to compare outcomes for OSA and non-OSA patient groups and according to EDS status.

Cox proportional hazards regression analysis was used to assess whether OSA was an independent prognostic factor for the observed events, with stratification for EDS status. Adjustments were made in the multivariable models for confounders that were clinically related to the outcomes, or showed an association with these endpoints in univariate analysis. The first model was constructed without any adjustments, while age and sex were covariates in the second model. The third model included variables from the second model, as well as body mass index (BMI), history of hypertension, hyperlipidemia, diabetes, current smoking, prior stroke, prior MI, and the clinical presentation including acute MI versus UA. All analyses were conducted by using SPSS, version 26.0 (IBM SPSS, Armonk, New York, NY, USA).

## 3. Results

### 3.1 Baseline and Clinical Characteristics

**Fig. 1.**
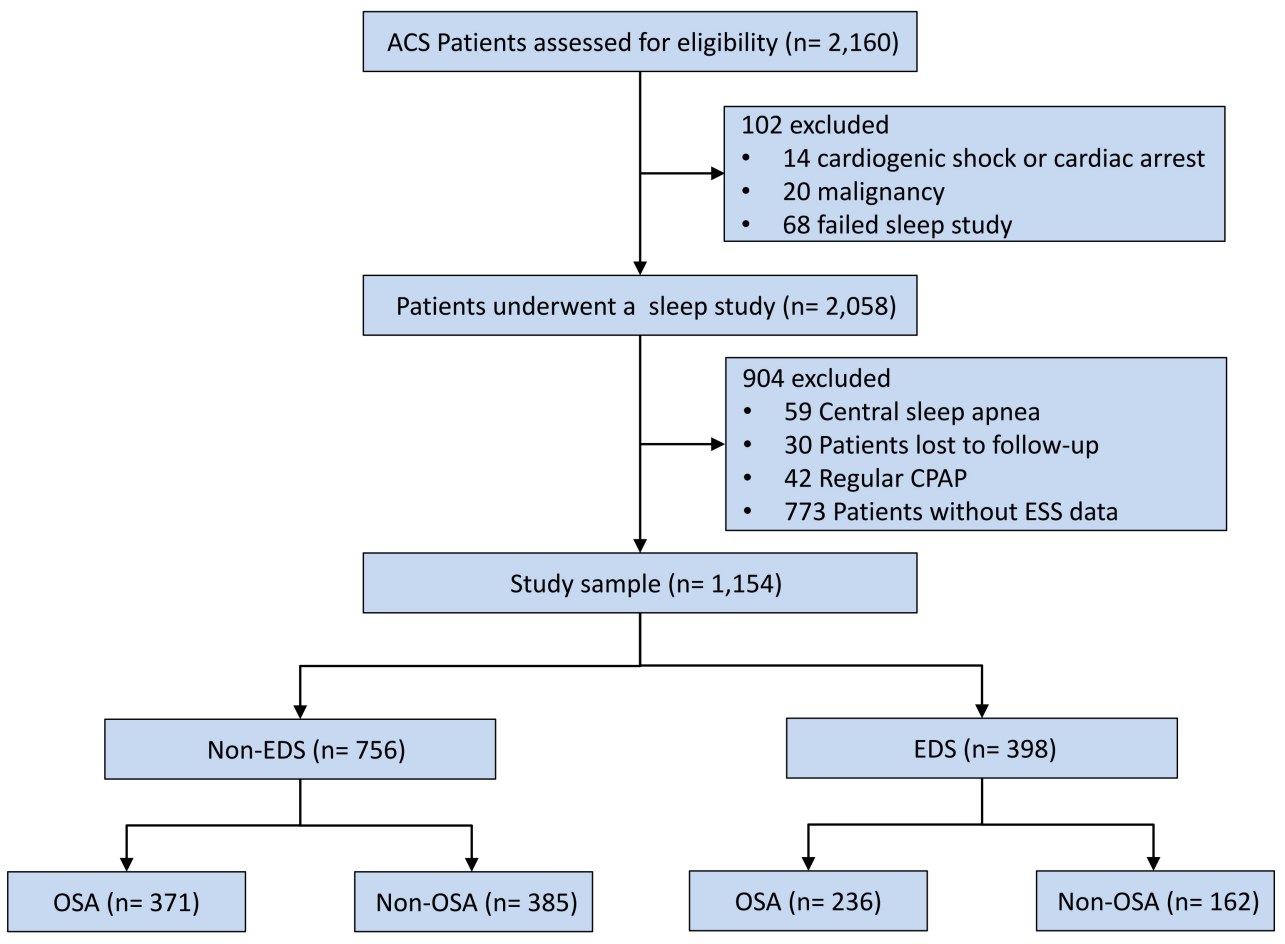
**Study flowchart**. Abbreviations: ACS, acute coronary syndrome; CPAP, continuous positive airway pressure; EDS, excessive daytime sleepiness; ESS, epworth sleepiness scale; OSA, obstructive sleep apnea.

The final analysis included 1154 patients (Fig. 1), of which 398 subjects (34.5%) had EDS and 756 (65.5%) did not have EDS. The EDS group had a greater percentage of males compared to the non-EDS group. EDS patients exhibited higher measures of obesity (BMI, waist and hip circumferences, ratio of waist/hip, neck circumference) and OSA (AHI, oxygen desaturation index [ODI], time with SaO2 <90%, ESS), along with elevated levels of triglycerides (**Supplementary Table 1**). A total of 607 (52.6%) patients had OSA. OSA was more common in the EDS group than in the non-EDS group (59.3% vs. 49.1%).

The measures of obesity and high-sensitivity C-reactive protein (hs-CRP) levels were elevated in the OSA group, both for EDS and non-EDS patient groups. No other differences were found in EDS patients. In non-EDS patients, those with OSA had a greater prevalence of hypertension, prior percutaneous coronary intervention (PCI), current drinking, a higher level of triglyceride, higher proportion of PCI, and larger number of stents placed compared to those without OSA (Table 1). Additionally, there is no difference in the primary baseline data and outcomes (Log rank *p* = 0.200) between excluded patients (n = 773) and included patients (n = 1154) (**Supplementary Table 4**, **Supplementary Fig. 2**).

**Table 1.** **Demographic and clinical characteristics in OSA versus non-OSA stratified by ESS categories**.

Variables		ESS ≥10 (n = 398)			ESS <10 (n = 756)		
		OSA (n = 236)	Non-OSA (n = 162)	*p*-value	OSA (n = 371)	Non-OSA (n = 385)	*p*-value
Demographics							
	Age, years	55.25 ± 10.60	56.1 ± 9.49	0.565	56.89 ± 11.14	56.66 ± 10.30	0.766
	Male	210 (89)	141 (87)	0.555	316 (85.2)	302 (78.4)	0.017
	BMI, kg/m^2^	28.52 ± 3.35	26.15 ± 3.54	<0.001	27.72 ± 3.68	25.74 ± 3.40	<0.001
	Waist circumference, cm	103.7 ± 10.22	96.77 ± 9.13	<0.001	101.81 ± 8.66	95.52 ± 8.7	<0.001
	Neck circumference, cm	42.05 ± 3.50	40.31 ± 2.97	<0.001	41.04 ± 3.21	39.27 ± 3.40	<0.001
	Hip circumference, cm	103.6 ± 7.39	99.81 ± 7.15	<0.001	102.35 ± 7.10	99.21 ± 6.57	<0.001
	Waist/hip ratio	1.00 (0.96–1.03)	0.97 (0.94–1.01)	0.001	0.99 (0.95–1.02)	0.97 (0.93–1.00)	<0.001
Medical history							
	Hypertension	158 (66.9)	103 (63.6)	0.487	252 (67.9)	224 (58.2)	0.006
	Hyperlipidemia	73 (30.9)	60 (37)	0.205	120 (32.3)	114 (29.6)	0.416
	Diabetes	76 (32.2)	55 (34)	0.716	107 (28.8)	107 (27.8)	0.949
	Prior PCI	44 (18.6)	36 (23.5)	0.244	95 (25.6)	63 (16.4)	0.002
	Prior stroke	31 (13.1)	18 (11.1)	0.546	40 (10.8)	38 (9.9)	0.680
	Prior MI	32 (13.6)	31 (19.1)	0.134	72 (19.4)	60 (15.6)	0.166
	Current smoking	119 (50.4)	78 (48.1)	0.656	176 (47.4)	157 (40.8)	0.065
	Current drinking	84 (35.6)	55 (34)	0.736	130 (35)	108 (28.1)	0.039
Laboratory data							
	Creatinine, µmol/L	74.7 (65.8–84.0)	73.3 (64.0–81.5)	0.222	73.7 (65.4–83.0)	70.8 (61.3–81.3)	0.019
	Hs-CRP, mg/L	2.64 (1.16–8.14)	1.50 (0.51–5.56)	0.001	2.44 (0.87–6.79)	1.41 (0.62–4.13)	<0.001
	LVEF, %	61 (56–65)	62 (56–66)	0.416	60 (55–65)	62 (58–65)	0.085
	LDL-C, mmol/L	2.55 (2.01–3.09)	2.36 (1.81–3.24)	0.358	2.4 (1.88–3.01)	2.43 (1.85–3.12)	0.644
	HDL-C, mmol/L	0.97 (0.86–1.11)	0.96 (0.85–1.16)	0.797	0.98 (0.84–1.14)	1.03 (0.88–1.22)	0.005
	TC, mmol/L	4.26 (3.62–4.88)	4.01 (3.35–5.19)	0.422	4.05 (3.45–4.81)	4.13 (3.42–4.98)	0.246
	TG, mmol/L	1.76 (1.26–2.40)	1.50 (1.15–2.32)	0.066	1.52 (1.12–2.14)	1.40 (1.04–2.02)	0.031
	HbA1c, %	6.2 (5.6–7.4)	5.9 (5.5–6.9)	0.089	6.1 (5.6–7.0)	6.0 (5.6–6.8)	0.113
	Systolic BP, mmHg	126 (119–138)	124 (112–135)	0.071	126 (117–139)	127 (118–139)	0.876
	Diastolic BP, mmHg	76 (70–86)	75 (67–80)	0.045	77 (70–85)	75 (69–83)	0.079
Sleep information							
	AHI, events per hr	33.45 (22.35–48.97)	7.95 (4.1–11.2)	<0.001	28.2 (20.1–41.6)	7.6 (4.2–10.3)	<0.001
	ODI, events per hr	30.6 (21.7–46.9)	8.8 (5.0–12.0)	<0.001	26.5 (19–38.5)	8.25 (4.92–11.60)	<0.001
	Minimum SaO2, %	82 (75–86)	87 (85–89)	<0.001	83 (77–86)	88 (85–90)	<0.001
	Mean SaO2, %	94 (93–95)	93 (92–94)	<0.001	93 (92–94)	94.3 (93–95)	<0.001
	Percentage of time with SaO2 <90%, %	8.0 (2.0–21.0)	1.0 (0.15–3.0)	<0.001	12.91 (2.0–17)	5.42 (0.10–3.15)	<0.001
	Epworth Sleepiness Scale	13.43 ± 2.9	12.88 ± 2.6	0.06	5.09 ± 2.9	4.42 ± 2.8	0.002
Diagnosis				0.448			0.273
	STEMI	60 (25.4)	38 (23.5)		85 (22.9)	70 (18.2)	
	NSTEMI	52 (22)	29 (17.9)		67 (18.1)	73 (19.0)	
	Unstable angina	124 (52.5)	95 (58.6)		219 (59.0)	242 (62.9)	
Non-obstructive CAD							
	MINOCA	6/112 (5.4)	3/67 (4.5)	0.781	5/152 (3.3)	11/143 (7.7)	0.092
	INOCA	12/124 (9.7)	9/95 (9.5)	0.963	19/219 (8.7)	19/242 (7.9)	0.775
Procedures							
	Coronary angiography	228 (96.6)	157 (96.9)	0.867	366 (98.7)	375 (97.4)	0.218
	PCI	137 (58.1)	83 (51.2)	0.449	211 (56.9)	187 (48.6)	0.164
	PTCA	21 (8.9)	13 (8.0)		31 (8.4)	35 (9.1)	
	CABG	12 (5.1)	15 (9.3)		23 (6.2)	36 (9.4)	
	Multivessel disease	153 (64.8)	107 (66)	0.968	242 (65.2)	221 (57.4)	0.082
	Number of stents	1 (0–1)	1 (0–1)	0.227	1 (0–2)	0 (0–1)	0.009
Medications on discharge							
	Aspirin	229 (97.0)	154 (95.1)	0.310	361 (97.3)	377 (97.9)	0.578
	P2Y_12_ inhibitor	219 (92.8)	149 (92.0)	0.760	342 (92.2)	347 (90.1)	0.321
	ACEI or ARB	153 (64.8)	95 (58.6)	0.211	236 (63.6)	216 (56.1)	0.035
	CCB	56 (23.7)	30 (18.5)	0.215	80 (21.6)	67 (17.4)	0.148
	β-blockers	185 (78.4)	124 (76.5)	0.664	298 (80.3)	286 (74.3)	0.048
	Statins	234 (99.2)	157 (96.9)	0.095	363 (97.8)	381 (99.0)	0.219

Abbreviations: ACEI, angiotensin-converting enzymes inhibitor; AHI, apnea-hypopnea index; ARB, angiotensin receptor blocker; BMI, body mass index; BP, blood pressure; CABG, coronary artery bypass grafting; CAD, coronary artery disease; CCB, calcium channel blockers; ESS, epworth sleepiness scale; HDL-C, high-density lipoprotein cholesterol; Hs-CRP, high-sensitivity C-reactive protein; INOCA, ischemia with non-obstructive coronary artery disease [defined as angina with non-obstructive CAD (<50% diameter stenosis)]; LVEF, left ventricular ejection fractions; LDL-C, low-density lipoprotein cholesterol; MI, myocardial infarction; MINOCA, myocardial infarction with non-obstructive coronary artery disease [defined as MI with non-obstructive CAD (<50% diameter stenosis)]; NSTEMI, non-ST-segment elevation myocardial infarction; ODI, oxygen desaturation index; OSA, obstructive sleep apnea; PCI, percutaneous coronary intervention; PTCA, percutaneous transluminal coronary angioplasty; SaO2, arterial oxygen saturation; STEMI, ST-segment-elevation myocardial infarction; TC, total cholesterol; TG, triglyceride; Data are presented as mean ± standard deviation, median (first quartile, third quartile), n (%).

### 3.2 Outcomes for EDS and non-EDS Groups

MACCEs occurred in 223 patients during the median follow-up period of 2.9 years (interquartile range: 1.5–3.6), accounting for 19.3% of the total cohort. The incidence of MACCEs was not significantly different between patients who did or did not have EDS (adjusted hazard ratio (HR) = 1.16, 95% CI: 0.89–1.52, *p* = 0.28) (**Supplementary Fig. 1**). For the secondary endpoints, cardiovascular death was observed in 22 patients (1.9%), MI in 31 patients (2.7%), stroke in 24 patients (2.1%), ischemia-driven revascularization in 133 patients (11.5%), hospitalization for UA in 156 patients (13.5%), and hospitalization for HF in 7 patients (0.6%). For the other individual cardiovascular events, no significant differences were observed between EDS and non-EDS patients (**Supplementary Table 3**).

### 3.3 Outcomes for OSA and Non-OSA Groups Stratified According to EDS Status

**Fig. 2.**
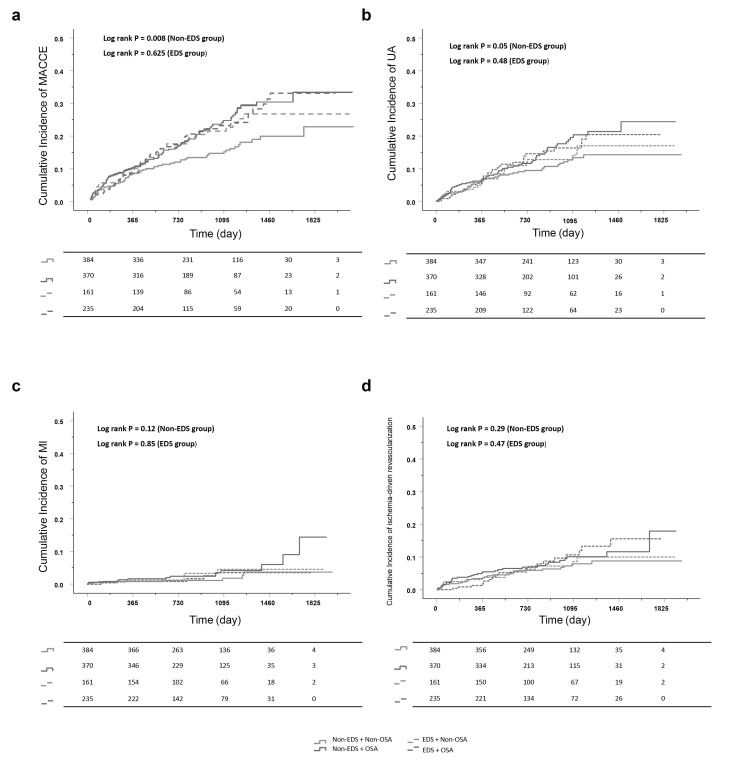
**Kaplan-Meier curves in OSA vs non-OSA groups stratified by EDS categories**. (a) MACCE, (b) hospitalization for unstable angina, (c) myocardial infarction, and (d) ischemia-driven revascularization. Abbreviations: MACCE, major adverse cardiovascular and cerebrovascular event; EDS, Excessive daytime sleepiness; MI, myocardial infarction; OSA, obstructive sleep apnea; UA, unstable angina.

We next categorized EDS patients into those with or without OSA, and non-EDS patients into those with or without OSA. For EDS patients, no significant difference in MACCEs was found between OSA and non-OSA groups (HR = 1.12, 95% CI: 0.72–1.72, *p* = 0.63). Moreover, the outcomes did not change after adjusting for clinical confounders (adjusted HR = 1.05, 95% CI: 0.67–1.66, *p* = 0.84). Similarly, in patients with EDS, no significant differences were found between OSA and non-OSA groups for any secondary endpoint. For patients without EDS, a significantly higher risk of MACCEs was observed in OSA patients compared to non-OSA patients (HR = 1.57, 95% CI: 1.12–2.20, *p* = 0.008). Following adjustment for clinical confounders, multivariable analysis revealed OSA was an independent predictor for MACCEs in patients without EDS (adjusted HR = 1.42, 95% CI: 1.01–2.02, *p* = 0.046). No significant interactions between EDS and OSA were observed for MACCEs (interaction *p* = 0.23). Notably, in patients without EDS, a difference in hospitalization for unstable angina was observed in model 1 (HR = 1.32, 95% CI: 0.99–2.22, *p* = 0.05). However, this difference was not significant in model 2 and model 3. No differences were observed in other secondary endpoints (Table 2, Fig. 2).

**Table 2.** **Cox regression analysis for clinical outcomes in OSA versus non-OSA stratified by ESS**.

Variables		ESS ≥10 (n = 398)		ESS <10 (n = 756)	
		HR (95% CI)	*p*-value	HR (95% CI)	*p*-value
MACCE					
	Model 1	1.12 (0.72–1.72)	0.63	1.57 (1.12–2.20)	0.008
	Model 2	1.13 (0.73–1.75)	0.60	1.52 (1.09–2.14)	0.015
	Model 3	1.05 (0.67–1.66)	0.84	1.42 (1.01–2.02)	0.046
Cardiovascular death					
	Model 1	0.87 (0.23–3.24)	0.84	1.72 (0.56–5.24)	0.34
	Model 2	0.90 (0.24–3.35)	0.87	1.62 (0.53–4.97)	0.40
	Model 3	0.71 (0.19–2.70)	0.62	1.68 (0.53–5.31)	0.38
Myocardial infarction					
	Model 1	0.89 (0.27–2.91)	0.85	2.08 (0.84–5.15)	0.12
	Model 2	0.89 (0.27–2.93)	0.85	1.87 (0.75–4.67)	0.18
	Model 3	0.91 (0.26–3.20)	0.89	1.43 (0.56–3.67)	0.45
Stroke					
	Model 1	0.86 (0.26–2.83)	0.81	0.92 (0.31–2.73)	0.88
	Model 2	0.88 (0.27–2.88)	0.83	0.90 (0.30–2.69)	0.85
	Model 3	0.94 (0.27–3.25)	0.93	0.84 (0.27–2.58)	0.76
Ischemia-driven revascularization					
	Model 1	1.30 (0.64–2.65)	0.47	1.32 (0.79–2.22)	0.29
	Model 2	1.32 (0.65–2.70)	0.44	1.26 (0.75–2.11)	0.39
	Model 3	1.20 (0.57–2.52)	0.64	1.13 (0.66–1.93)	0.66
Hospitalization for unstable angina					
	Model 1	1.22 (0.71–2.09)	0.48	1.47 (0.99–2.18)	0.05
	Model 2	1.23 (0.72–2.11)	0.45	1.43 (0.96–2.13)	0.08
	Model 3	1.13 (0.64–1.99)	0.67	1.37 (0.91–2.06)	0.14
Hospitalization for heart failure					
	Model 1	0.75 (0.05–12.1)	0.84	1.58 (0.27–9.48)	0.61
	Model 2	0.68 (0.04–11.3)	0.79	1.38 (0.23–8.25)	0.73
	Model 3	0.23 (0.01–5.67)	0.37	1.18 (0.19–7.51)	0.86

Abbreviations: CI, confidence interval; ESS, epworth sleepiness scale; HR, hazard ratio; MACCE, major adverse cardiovascular and cerebrovascular event. Model 1: Unadjusted model; Model 2: Adjusted for age, sex; Model 3: Adjusted for age, sex, body mass index, current smoking, history of hypertension, diabetes, dyslipidemia, prior myocardial infarction, prior stroke, and clinical presentation (acute myocardial infarction vs unstable angina).

Finally, we performed sensitivity analysis to assess stability of the effect on the primary endpoint in patients without EDS. The observed association between OSA and MACCE showed no influence from confounding factors (Table 3).

**Table 3.** **Sensitivity analysis of MACCE in OSA vs. Non-OSA in patients without EDS**.

Subgroups		HR (95% CI)	*p*-value	*p*-value for interaction
Old age				0.257
	Yes	2.199 (1.108–4.363)	0.024	
	No	1.389 (0.939–2.055)	0.100	
Sex				0.897
	Male	1.522 (1.053–2.201)	0.026	
	Female	1.675 (0.709–3.956)	0.239	
Obesity				0.448
	Yes	1.931 (0.982–3.797)	0.057	
	No	1.408 (0.936–2.12)	0.101	
Diabetes				0.120
	Yes	2.297 (1.282–4.116)	0.005	
	No	1.287 (0.846–1.957)	0.239	
Dyslipidemia				0.306
	Yes	2.123 (1.116–4.036)	0.022	
	No	1.393 (0.934–2.079)	0.104	
Hypertension				0.714
	Yes	1.621 (1.069–2.459)	0.023	
	No	1.402 (0.781–2.516)	0.257	
Diagnosis				0.743
	STEMI	1.568 (0.793–3.098)	0.196	
	NSTEMI	1.145 (0.566–2.320)	0.706	
	UA	1.765 (1.102–2.828)	0.018	

Abbreviations: HR, hazard ratio; NSTEMI, non-ST-segment elevation myocardial infarction; STEMI, ST-segment elevation myocardial infarction; UA, unstable angina. Data are presented as median (first quartile, third quartile).

## 4. Discussion

For ACS patients without EDS, OSA was found to be associated with subsequent adverse cardiovascular events. However, this association was not observed for patients with EDS. Additionally, subgroup analyses revealed no differences in the non-EDS OSA group, indicating that OSA was a key predictor of MACCEs in patients with no EDS. These results advance our understanding in this field, as previous research suggested that EDS was associated with poor prognosis. Furthermore, our findings suggest that more attention should be given to OSA patients without EDS, as they may have an elevated risk of adverse events that has been overlooked in the past.

An earlier study indicated that EDS was associated with a higher incidence of MACE during a 4-year follow-up period in post-MI patients with moderate to severe OSA [13]. This relationship has been consistently observed in a wider cohort, including non-MI patients [25, 26, 27]. It suggests there may be an underlying pathophysiological mechanism that connects sleep disorder with cardiovascular risks, such as intermittent hypoxia, systemic inflammation, and other metabolic disorders [28]. However, some studies have reported different findings. Longitudinal analysis of participants in the Nurses’ Health Study II found that daytime sleepiness was not independently associated with the risk of cardiovascular disease [29]. Another recent study based on the UK Biobank database revealed that EDS was not a significant risk factor for incident MI or stroke, regardless of sleep duration [30]. The focus of our study was patients with established ACS. An increased risk of subsequent MACCEs was observed in OSA patients with no EDS, but not in those with EDS. However, the small size of each group and low frequency of events reduced the statistical power of the study. Hence, caution is required in interpreting the results, and additional trials with large cohorts are needed to validate our results.

To minimize the potential bias, patients with regular CPAP were excluded from this analysis. However, from an ethical perspective, OSA patients with severe daytime sleepiness should be treated with CPAP. Notably, previous randomized controlled trials on patients with cardiovascular disease did not demonstrate a benefit from CPAP on the incidence of cardiovascular events [31, 32]. Post hoc analysis indicated that patients who adhered to CPAP therapy (≥4 h per night) had better outcomes than those who did not receive CPAP, or who used it for <4 h per night [33]. Our findings highlight that OSA patients have an increased cardiovascular risk, even without EDS. Therefore, intervention for OSA should not be completely neglected with this phenotype. It may not be appropriate to completely reject any intervention for OSA patients without EDS. Nevertheless, the challenge of maintaining CPAP adherence and the potential impact of patient characteristics on treatment outcomes should not be ignored. Indeed, CPAP is not the only intervention option for OSA. Given the emphasis on the heterogeneity of OSA and baseline characteristics, improving the ‘personalization’ of OSA therapy and identifying high-risk indications for potential benefit may be the best option. Weight loss regimen in concert with CPAP therapy reduced cardiovascular risk factor compared with either intervention alone [34]. Beneficial effects on blood pressure have also been shown with the use of devices for mandibular advancement [35].

Potential associations between OSA and clinical outcomes in patients without EDS may be better understood by analyzing the unique characteristics of OSA symptoms, pathophysiological mechanisms, and their consequences. Exploring endocrine dysfunction, inflammation, sympathetic hyperactivity, and oxidative stress in sleep-deprived individuals may clarify the mechanisms linking non-EDS and cardiovascular outcomes in ACS patients. EDS is closely associated with established factors for cardiovascular risk, including obesity, uncontrolled hypertension, diabetes, and a sedentary lifestyle [36, 37]. Notably, while our findings indicated that OSA had a more pronounced impact on the prognosis of non-sleepy patients compared to those of sleepy patients, EDS per se still had an adverse trend (although not statistically significant) on the prognosis. This observation aligns with the study focused on the symptom subtypes, which reported that patients with the excessively sleepy subtype were at increased risk of cardiovascular disease compared to patients with minimally or moderately sleepy [27].

OSA correlates with a higher prevalence of hypertension, HF, diabetes, and atrial fibrillation. Even in patients without EDS, those with OSA tend to have a poor prognosis. The obstructive respiratory episodes characteristic of OSA may induce circadian dysregulation, primarily mediated by sleep disorder and intermittent hypoxia. This disruption triggers inflammatory responses and disturbs neural and hormonal balance, ultimately leading to dysregulation of the molecular circadian clock and associated biological pathways [38]. Prolonged exposure to hypoxia is implicated in the development of disorders commonly associated with circadian rhythm disturbances, including cardiovascular and respiratory diseases, dementia, cancer, and metabolic disorders [39, 40]. Several studies have also demonstrated that OSA patients exhibit significantly higher sympathetic nerve activity in postganglionic muscles during wakefulness and normal breathing patterns [41, 42]. Additionally, patients with OSA have increased basal sympathetic tone during wakefulness, and experience acute cyclical sympathetic excitation during sleep [43]. We hypothesize that sympathetic excitation in OSA patients without EDS may further contribute to poor prognosis. Additional research is needed to investigate such mechanisms.

The present study has a number of limitations. Firstly, Type III portable polygraphy was used to diagnose OSA. This could underestimate the severity of OSA, as the total sleep time cannot be calculated accurately. Nevertheless, portable polygraphy is a viable alternative to polysomnography for diagnosing OSA [44]. Second, OSA severity in the acute setting of ACS may have been overestimated [45]. To minimize this potential bias, clinical stabilization was achieved prior to conducting the sleep study during hospitalization. Third, because of its subjectivity the assessment of daytime sleepiness was limited to a one-time questionnaire. Daytime sleepiness is variable and may not be accurately captured by a single measurement. Fourth, about 40% of patients were excluded due to unavailable ESS, which may lead to selection bias. But there was no difference in the primary baseline data and outcomes between excluded patients and included patients, which may allay some of the concerns of selection bias. Fifth, our study was that the majority of participants were of Asian population. This demographic characteristic may limit the generalizability of our findings to other ethnic populations. Sixth, this cohort showed a high proportion of UA and a low proportion of revascularization, suggesting that enrolled ACS patients were relatively at lower risk. This may introduce a potential bias and the results need to be further validated in high-risk populations.

## 5. Conclusions

A higher risk of MACCEs was observed in ACS patients with OSA but with no EDS. This finding highlights the necessity for comprehensive OSA screening of all ACS patients, regardless of their EDS status. Moreover, this study also underlines the importance of intervention in OSA patients without EDS.

## Data Availability

The datasets used and analyzed during the current study are available from the corresponding author on reasonable request.

## Abbreviations

ACEI, angiotensin-converting enzymes inhibitor; ACS, acute coronary syndrome; AHI, apnea-hypopnea index; ARB, angiotensin receptor blocker; BMI, body mass index; BP, blood pressure; CABG, coronary artery bypass grafting; CAD, coronary artery disease; CCB, calcium channel blockers; CPAP, continuous positive airway pressure; EDS, excessive daytime sleepiness; ESS, Epworth Sleepiness Scale; HDL-C, high-density lipoprotein cholesterol; HF, heart failure; HR, hazard ratio; Hs-CRP, high-sensitivity C-reactive protein; INOCA, ischemia with non-obstructive coronary artery disease [defined as angina with non-obstructive CAD (<50% diameter stenosis)]; LVEF, left ventricular ejection fractions; LDL-C, low-density lipoprotein cholesterol; MACE, major adverse cardiovascular events; MACCE, major adverse cardiac and cerebrovascular events; MI, myocardial infarction; MINOCA, myocardial infarction with non-obstructive coronary artery disease [defined as MI with non-obstructive CAD (<50% diameter stenosis)]; NSTEMI, non-ST-segment elevation myocardial infarction; ODI, oxygen desaturation index; OSA, obstructive sleep apnea; PCI, percutaneous coronary intervention; PTCA, percutaneous transluminal coronary angioplasty; SaO2, oxygen saturation; STEMI, ST-segment-elevation myocardial infarction; TC, total cholesterol; TG, triglyceride; UA, unstable angina.

## Supplementary Material

Supplementary material associated with this article can be found, in the online version, at https://doi.org/10.31083/RCM33439.

## References

[1] Benjafield AV, Ayas NT, Eastwood PR, Heinzer R, Ip MSM, Morrell MJ (2019). Estimation of the global prevalence and burden of obstructive sleep apnoea: a literature-based analysis. *Respiratory Medicine*.

[2] Koo CY, de la Torre AS, Loo G, Torre MSDL, Zhang J, Duran-Cantolla J (2017). Effects of Ethnicity on the Prevalence of Obstructive Sleep Apnoea in Patients with Acute Coronary Syndrome: A Pooled Analysis of the ISAACC Trial and Sleep and Stent Study. *Heart, Lung & Circulation*.

[3] Leão S, Conde B, Fontes P, Calvo T, Afonso A, Moreira I (2016). Effect of Obstructive Sleep Apnea in Acute Coronary Syndrome. *The American Journal of Cardiology*.

[4] Randerath W, Bonsignore MR, Herkenrath S (2019). Obstructive sleep apnoea in acute coronary syndrome. *European Respiratory Review: an Official Journal of the European Respiratory Society*.

[5] Hao W, Wang X, Fan J, Guo R, Gong W, Yan Y (2023). Prognostic Implications of OSA in Acute Coronary Syndrome by Obesity Status. *Chest*.

[6] Zapater A, Sánchez-de-la-Torre M, Benítez ID, Targa A, Bertran S, Torres G (2020). The Effect of Sleep Apnea on Cardiovascular Events in Different Acute Coronary Syndrome Phenotypes. *American Journal of Respiratory and Critical Care Medicine*.

[7] Zapater A, Solelhac G, Sánchez-de-la-Torre A, Gracia-Lavedan E, Benitez ID, Torres G (2022). Respiratory Polygraphy Patterns and Risk of Recurrent Cardiovascular Events in Patients With Acute Coronary Syndrome. *Frontiers in Medicine*.

[8] Stepnowsky C, Sarmiento KF, Bujanover S, Villa KF, Li VW, Flores NM (2019). Comorbidities, Health-Related Quality of Life, and Work Productivity Among People With Obstructive Sleep Apnea With Excessive Sleepiness: Findings From the 2016 US National Health and Wellness Survey. *Journal of Clinical Sleep Medicine*.

[9] Mulgrew AT, Ryan CF, Fleetham JA, Cheema R, Fox N, Koehoorn M (2007). The impact of obstructive sleep apnea and daytime sleepiness on work limitation. *Sleep Medicine*.

[10] Lal C, Weaver TE, Bae CJ, Strohl KP (2021). Excessive Daytime Sleepiness in Obstructive Sleep Apnea. Mechanisms and Clinical Management. *Annals of the American Thoracic Society*.

[11] Xiong Y, Zhou XJ, Nisi RA, Martin KR, Karaman MM, Cai K (2017). Brain white matter changes in CPAP-treated obstructive sleep apnea patients with residual sleepiness. *Journal of Magnetic Resonance Imaging*.

[12] Joo EY, Tae WS, Lee MJ, Kang JW, Park HS, Lee JY (2010). Reduced brain gray matter concentration in patients with obstructive sleep apnea syndrome. *Sleep*.

[13] Xie J, Sert Kuniyoshi FH, Covassin N, Singh P, Gami AS, Chahal CAA (2018). Excessive Daytime Sleepiness Independently Predicts Increased Cardiovascular Risk After Myocardial Infarction. *Journal of the American Heart Association*.

[14] Thygesen K, Alpert JS, Jaffe AS, Simoons ML, Chaitman BR, White HD (2012). Third universal definition of myocardial infarction. *European Heart Journal*.

[15] Thygesen K, Alpert JS, Jaffe AS, Chaitman BR, Bax JJ, Morrow DA (2019). Fourth universal definition of myocardial infarction (2018). *European Heart Journal*.

[16] Anderson JL, Adams CD, Antman EM, Bridges CR, Califf RM, Casey DE (2007). ACC/AHA 2007 guidelines for the management of patients with unstable angina/non-ST-Elevation myocardial infarction: a report of the American College of Cardiology/American Heart Association Task Force on Practice Guidelines (Writing Committee to Revise the 2002 Guidelines for the Management of Patients With Unstable Angina/Non-ST-Elevation Myocardial Infarction) developed in collaboration with the American College of Emergency Physicians, the Society for Cardiovascular Angiography and Interventions, and the Society of Thoracic Surgeons endorsed by the American Association of Cardiovascular and Pulmonary Rehabilitation and the Society for Academic Emergency Medicine. *Journal of the American College of Cardiology*.

[17] Sateia MJ (2014). International classification of sleep disorders-third edition: highlights and modifications. *Chest*.

[18] Epstein LJ, Kristo D, Strollo PJ, Friedman N, Malhotra A, Patil SP (2009). Clinical guideline for the evaluation, management and long-term care of obstructive sleep apnea in adults. *Journal of Clinical Sleep Medicine*.

[19] Sánchez-de-la-Torre M, Sánchez-de-la-Torre A, Bertran S, Abad J, Duran-Cantolla J, Cabriada V (2020). Effect of obstructive sleep apnoea and its treatment with continuous positive airway pressure on the prevalence of cardiovascular events in patients with acute coronary syndrome (ISAACC study): a randomised controlled trial. *The Lancet. Respiratory Medicine*.

[20] Ibanez B, James S, Agewall S, Antunes MJ, Bucciarelli-Ducci C, Bueno H (2018). 2017 ESC Guidelines for the management of acute myocardial infarction in patients presenting with ST-segment elevation: The Task Force for the management of acute myocardial infarction in patients presenting with ST-segment elevation of the European Society of Cardiology (ESC). *European Heart Journal*.

[21] Roffi M, Patrono C, Collet JP, Mueller C, Valgimigli M, Andreotti F (2016). 2015 ESC Guidelines for the management of acute coronary syndromes in patients presenting without persistent ST-segment elevation: Task Force for the Management of Acute Coronary Syndromes in Patients Presenting without Persistent ST-Segment Elevation of the European Society of Cardiology (ESC). *European Heart Journal*.

[22] Johns MW (1991). A new method for measuring daytime sleepiness: the Epworth sleepiness scale. *Sleep*.

[23] Wang X, Fan J, Guo R, Hao W, Gong W, Yan Y (2023). Association of obstructive sleep apnoea with cardiovascular events in women and men with acute coronary syndrome. *The European Respiratory Journal*.

[24] Hicks KA, Tcheng JE, Bozkurt B, Chaitman BR, Cutlip DE, Farb A (2015). 2014 ACC/AHA Key Data Elements and Definitions for Cardiovascular Endpoint Events in Clinical Trials: A Report of the American College of Cardiology/American Heart Association Task Force on Clinical Data Standards (Writing Committee to Develop Cardiovascular Endpoints Data Standards). *Circulation*.

[25] Lee CH, Ng WY, Hau W, Ho HH, Tai BC, Chan MY (2013). Excessive daytime sleepiness is associated with longer culprit lesion and adverse outcomes in patients with coronary artery disease. *Journal of Clinical Sleep Medicine*.

[26] Empana JP, Dauvilliers Y, Dartigues JF, Ritchie K, Gariepy J, Jouven X (2009). Excessive daytime sleepiness is an independent risk indicator for cardiovascular mortality in community-dwelling elderly: the three city study. *Stroke*.

[27] Mazzotti DR, Keenan BT, Lim DC, Gottlieb DJ, Kim J, Pack AI (2019). Symptom Subtypes of Obstructive Sleep Apnea Predict Incidence of Cardiovascular Outcomes. *American Journal of Respiratory and Critical Care Medicine*.

[28] Gottlieb DJ, Punjabi NM (2020). Diagnosis and Management of Obstructive Sleep Apnea: A Review. *JAMA*.

[29] Gangwisch JE, Rexrode K, Forman JP, Mukamal K, Malaspina D, Feskanich D (2014). Daytime sleepiness and risk of coronary heart disease and stroke: results from the Nurses’ Health Study II. *Sleep Medicine*.

[30] Goodman MO, Dashti HS, Lane JM, Windred DP, Burns A, Jones SE (2023). Causal Association Between Subtypes of Excessive Daytime Sleepiness and Risk of Cardiovascular Diseases. *Journal of the American Heart Association*.

[31] Sánchez-de-la-Torre M, Gracia-Lavedan E, Benitez ID, Sánchez-de-la-Torre A, Moncusí-Moix A, Torres G (2023). Adherence to CPAP Treatment and the Risk of Recurrent Cardiovascular Events: A Meta-Analysis. *JAMA*.

[32] McEvoy RD, Antic NA, Heeley E, Luo Y, Ou Q, Zhang X (2016). CPAP for Prevention of Cardiovascular Events in Obstructive Sleep Apnea. *The New England Journal of Medicine*.

[33] Eulenburg C, Celik Y, Redline S, Thunström E, Glantz H, Strollo PJ (2023). Cardiovascular Outcomes in Adults with Coronary Artery Disease and Obstructive Sleep Apnea with versus without Excessive Daytime Sleepiness in the RICCADSA Cinical Trial. *Annals of the American Thoracic Society*.

[34] Koskinas KC, Van Craenenbroeck EM, Antoniades C, Blüher M, Gorter TM, Hanssen H (2025). Obesity and cardiovascular disease: an ESC clinical consensus statement. *European Journal of Preventive Cardiology*.

[35] Ou YH, Colpani JT, Cheong CS, Loke W, Thant AT, Shih EC (2024). Mandibular Advancement vs CPAP for Blood Pressure Reduction in Patients With Obstructive Sleep Apnea. *Journal of the American College of Cardiology*.

[36] Calhoun SL, Vgontzas AN, Fernandez-Mendoza J, Mayes SD, Tsaoussoglou M, Basta M (2011). Prevalence and risk factors of excessive daytime sleepiness in a community sample of young children: the role of obesity, asthma, anxiety/depression, and sleep. *Sleep*.

[37] Mbatchou Ngahane BH, Nganda MM, Dzudie A, Luma H, Kamdem F, Ngote HR (2015). Prevalence and determinants of excessive daytime sleepiness in hypertensive patients: a cross-sectional study in Douala, Cameroon. *BMJ Open*.

[38] Šmon J, Kočar E, Pintar T, Dolenc-Grošelj L, Rozman D (2023). Is obstructive sleep apnea a circadian rhythm disorder?. *Journal of Sleep Research*.

[39] Chen PS, Chiu WT, Hsu PL, Lin SC, Peng IC, Wang CY (2020). Pathophysiological implications of hypoxia in human diseases. *Journal of Biomedical Science*.

[40] Nisar A, Khan S, Li W, Hu L, Samarawickrama PN, Gold NM (2024). Hypoxia and aging: molecular mechanisms, diseases, and therapeutic targets. *MedComm*.

[41] Kasai T, Bradley TD (2011). Obstructive sleep apnea and heart failure: pathophysiologic and therapeutic implications. *Journal of the American College of Cardiology*.

[42] Somers VK, Dyken ME, Clary MP, Abboud FM (1995). Sympathetic neural mechanisms in obstructive sleep apnea. *The Journal of Clinical Investigation*.

[43] Taranto Montemurro L, Floras JS, Millar PJ, Kasai T, Gabriel JM, Spaak J (2012). Inverse relationship of subjective daytime sleepiness to sympathetic activity in patients with heart failure and obstructive sleep apnea. *Chest*.

[44] Corral J, Sánchez-Quiroga MÁ, Carmona-Bernal C, Sánchez-Armengol Á, de la Torre AS, Durán-Cantolla J (2017). Conventional Polysomnography Is Not Necessary for the Management of Most Patients with Suspected Obstructive Sleep Apnea. Noninferiority, Randomized Controlled Trial. *American Journal of Respiratory and Critical Care Medicine*.

[45] Buchner S, Greimel T, Hetzenecker A, Luchner A, Hamer OW, Debl K (2012). Natural course of sleep-disordered breathing after acute myocardial infarction. *The European Respiratory Journal*.

