# Supplementary material for: Association of Obstructive Sleep Apnea With Cardiovascular Events in Acute Coronary Syndrome Patients With or Without Excessive Daytime Sleepiness: A Prospective Cohort Study
Source: Rev Cardiovasc Med. 2025 Jul 28;26(7):33439. doi: 10.31083/RCM33439 (PMC12326451; doi:10.31083/RCM33439)
Supplement: Supplementary file 1 [file 2153-8174-26-7-33439-s1.zip › Supplementary Material.docx]

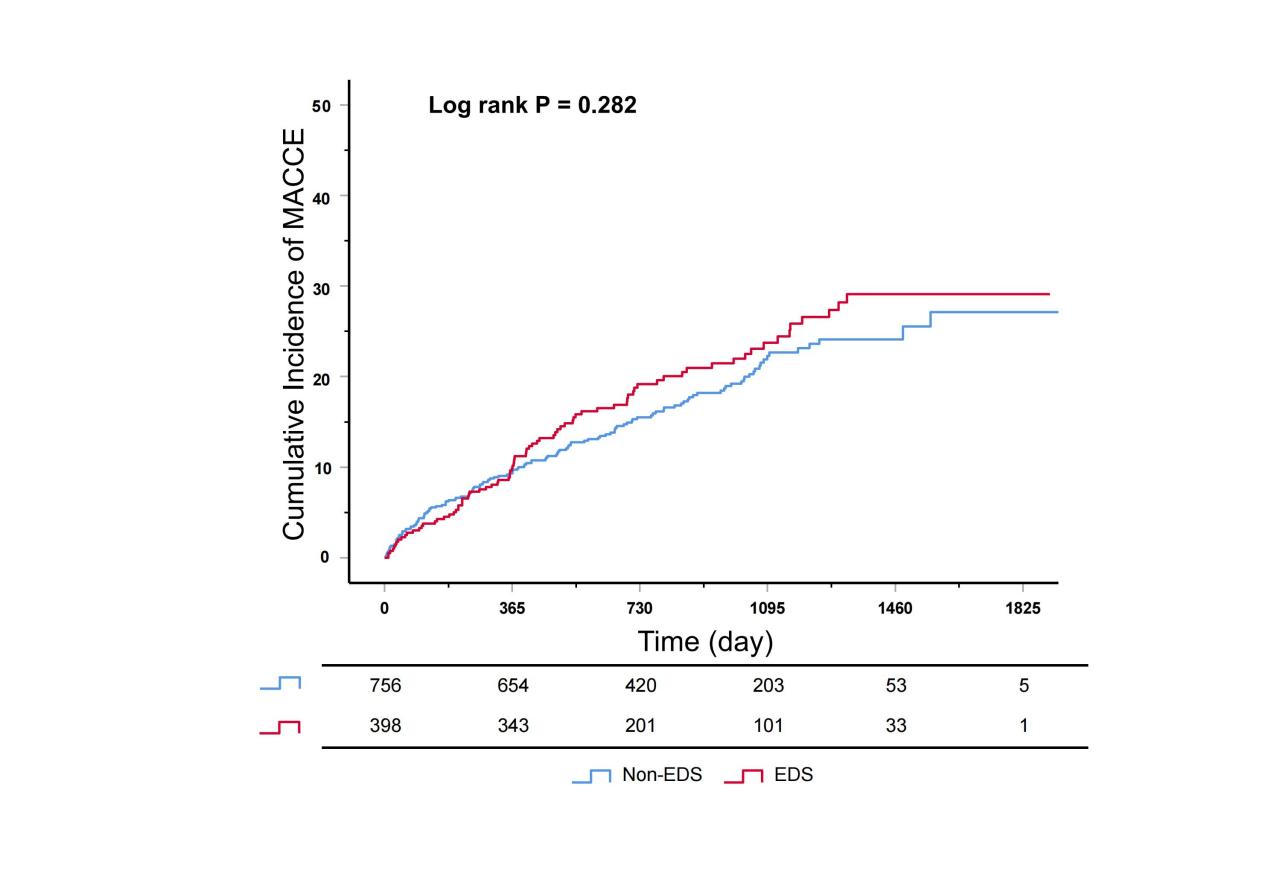


Supplementary Fig. 1. Kaplan-Meier curves for MACCE in ACS patients with EDS or without EDS. Abbreviations: ACS, acute coronary syndrome; MACCE, major adverse cardiovascular and cerebrovascular event; EDS, Excessive daytime sleepiness; OSA, obstructive sleep apnea.

Supplementary Table 1. Clinical outcomes in OSA and non-OSA stratified by ESS

| Variables | ALL (n=1154) | ESS≥10 (n=398) | | ESS＜10 (n=756) | |
| --- | --- | --- | --- | --- | --- |
|  |  | OSA (n=236) | Non-OSA (n=162) | OSA (n=371) | Non-OSA (n=385) |
| MACCE | 223 | 51 | 33 | 81 | 58 |
| Cardiovascular death | 22 | 5 | 4 | 8 | 5 |
| Myocardial infarction | 31 | 5 | 5 | 14 | 7 |
| Stroke | 24 | 6 | 5 | 6 | 7 |
| Ischemia-driven revascularization | 133 | 30 | 20 | 48 | 35 |
| Hospitalization for unstable angina | 156 | 35 | 21 | 57 | 43 |
| Hospitalization for heart failure | 7 | 1 | 1 | 3 | 2 |

Abbreviations: MACCE, major adverse cardiovascular and cerebrovascular event.

Supplementary Table 2. Demographic and clinical characteristics in EDS versus non-EDS

| Variables | ESS≥10 (n=398) | ESS＜10 (n=756) | p-value |
| --- | --- | --- | --- |
| Demographics |  |  |  |
| Age, years | 55.59 ± 10.16 | 56.77 ± 10.71 | 0.071 |
| Male | 351 (88.2) | 618 (81.7) | 0.005 |
| BMI, kg/m2 | 27.55 ± 3.62 | 26.72 ± 3.55 | 0.000 |
| Waist circumference, cm | 100.88 ± 10.36 | 98.58 ± 9.11 | 0.000 |
| Neck circumference, cm | 41.33 ± 3.40 | 40.14 ± 3.43 | 0.000 |
| Hip circumference, cm | 102.08 ± 7.53 | 100.74 ± 7.01 | 0.003 |
| Waist/hip ratio | 0.99 (0.95-1.02) | 0.98 (0.94-1.01) | 0.008 |
| Medical history |  |  |  |
| Hypertension | 261 (65.6) | 476 (63) | 0.379 |
| Hyperlipidemia | 133 (33.4) | 234 (31) | 0.393 |
| Diabetes | 131 (32.9) | 214 (28.3) | 0.104 |
| Prior PCI | 80 (20.1) | 158 (20.9) | 0.906 |
| Prior stroke | 49 (12.3) | 78 (10.3) | 0.304 |
| Prior MI | 63 (15.8) | 132 (17.5) | 0.482 |
| Current smoking | 197 (49.5) | 333 (44) | 0.077 |
| Current drinking | 139 (34.9) | 238 (31.5) | 0.236 |
| Laboratory data |  |  |  |
| Creatinine (μmol/L) | 74.1 (65.4-83.5) | 71.85 (63.17-82.2) | 0.044 |
| Hs-CRP (mg/L) | 2.23 (0.86-8.28) | 1.96 (0.70-5.98) | 0.158 |
| LVEF, % | 62 (56-65) | 61 (56-65) | 0.995 |
| LDL-C, mmol/L | 2.48 (1.94-3.16) | 2.41 (1.86-3.08) | 0.123 |
| HDL-C, mmol/L | 0.96 (0.86-1.13) | 1.01 (0.86-1.18) | 0.019 |
| TC, mmol/L | 4.20 (3.5-5.01) | 4.08 (3.45-4.91) | 0.223 |
| TG, mmol/L | 1.68 (1.23-2.39) | 1.47 (1.07-2.08) | 0.000 |
| HbA1c (%) | 6.1 (5.6-7.2) | 6.0 (5.6-6.9) | 0.198 |
| Systolic BP (mmHg) | 126 (116-138) | 127 (117-139) | 0.461 |
| Diastolic BP (mmHg) | 75 (70-84) | 76 (70-84) | 0.626 |
| Sleep information |  |  |  |
| AHI (events per hr) | 19.5 (9.3-37.58) | 14.6 (7.5-27.68) | 0.000 |
| ODI (events per hr) | 19 (10.15-34.42) | 14.5 (8.1-26.4) | 0.000 |
| Minimum SaO2 (%) | 85 (80-88) | 86 (82-89) | 0.000 |
| Mean SaO2 (%) | 94 (92-95) | 94 (93-95) | 0.001 |
| Time with SaO2 < 90% (%) | 3 (0.9-13) | 2 (0.3-8.9) | 0.001 |
| Epworth Sleepiness Scale | 13.21 ± 2.8 | 4.75 ± 2.8 | 0.000 |
| Diagnosis |  |  | 0.132 |
| STEMI | 98 (24.6) | 155 (20.5) |  |
| NSTEMI | 81 (20.4) | 140 (18.5) |  |
| Unstable angina | 219 (55) | 461 (61) |  |
| Non-obstructive CAD |  |  |  |
| MINOCA | 9/179 (5.0) | 16/295 (5.4) | 0.862 |
| INOCA | 21/219 (9.6) | 38/461 (8.2) | 0.503 |
| Procedures |  |  |  |
| Coronary angiography | 385 (96.7) | 741 (98) | 0.178 |
| PCI | 220 (55.3) | 398 (52.6) | 0.585 |
| PTCA | 34 (8.5) | 66 (8.7) |  |
| CABG | 27 (6.8) | 59 (7.8) |  |
| Multivessel disease | 260 (65.3) | 463 (61.2) | 0.130 |
| Number of stents | 1 (0-1) | 1 (0-1) | 0.763 |
| Medications on discharge |  |  |  |
| Aspirin | 383 (96.2) | 738 (97.6) | 0.179 |
| P2Y12 inhibitor | 368 (92.5) | 689 (91.1) | 0.441 |
| ACEI or ARB | 248 (62.3) | 452 (59.8) | 0.404 |
| CCB | 86 (21.6) | 147 (19.4) | 0.384 |
| β-blockers | 309 (77.6) | 584 (77.2) | 0.880 |
| Statins | 391 (98.2) | 744 (98.4) | 0.828 |

Abbreviations: ACEI, angiotensin-converting enzymes inhibitor; AHI, apnea-hypopnea index; ARB, angiotensin receptor blocker; BMI, body mass index; BP, blood pressure; CABG, coronary artery bypass grafting; CCB, calcium channel blockers; EDS, Excessive daytime sleepiness; ESS, epworth sleepiness scale; HDL-C, high-density lipoprotein cholesterol; Hs-CRP, high-sensitivity C-reactive protein; INOCA, ischemia with non-obstructive coronary artery disease [defined as angina with non-obstructive CAD (<50% diameter stenosis)]; LVEF, left ventricular ejection fractions; LDL-C, low-density lipoprotein cholesterol; MINOCA, myocardial infarction with non-obstructive coronary artery disease [defined as MI with non-obstructive CAD (<50% diameter stenosis)]; NSTEMI, non-ST-segment elevation myocardial infarction; ODI, oxygen desaturation index; OSA, obstructive sleep apnea; PCI, percutaneous coronary intervention; PTCA, percutaneous transluminal coronary angioplasty; SaO2, arterial oxygen saturation; STEMI, ST-segment-elevation myocardial infarction; TC, total cholesterol; TG, triglyceride.

Data are presented as mean ± standard deviation, median (first quartile, third quartile), n (%).

Supplementary Table 3. Clinical outcomes in EDS versus non-EDS groups

| Variables | HR (95%CI) | p-value |
| --- | --- | --- |
| MACCE | 1.160 (0.885-1.522) | 0.282 |
| Cardiovascular death | 1.298 (0.554-3.041) | 0.547 |
| Myocardial infarction | 0.982 (0.473-2.039) | 0.961 |
| Stroke | 1.614 (0.723-3.604) | 0.243 |
| Ischemia-driven revascularization | 1.068 (0.697-1.638) | 0.762 |
| Hospitalization for unstable angina | 1.072 (0.773-1.487) | 0.677 |
| Hospitalization for heart failure | 0.689 (0.133-3.567) | 0.657 |

Abbreviations: CI, confidence interval; HR, hazard ratio; MACCE, major adverse cardiovascular and cerebrovascular event.

Data are presented as median (first quartile, third quartile).

Supplementary Table 4. Demographic and clinical characteristics of included and excluded patients

| Variables | Included patients (n=1154) | Excluded patients (n=773) | p-value |
| --- | --- | --- | --- |
| Demographics |  |  |  |
| Age, years | 56.3 ± 10.5 | 56.4 ± 10.4 | 0.951 |
| Male | 969 (84.0) | 660 (85.4) | 0.401 |
| BMI, kg/m^2^ | 27.0 ± 3.6 | 27.2 ± 3.7 | 0.339 |
| Waist circumference, cm | 99.4 ± 9.6 | 99.8 ± 9.5 | 0.328 |
| Neck circumference, cm | 40.5 ± 3.5 | 40.7 ± 4.0 | 0.236 |
| Hip circumference, cm | 101.2 ± 7.2 | 101.4 ± 7.3 | 0.540 |
| Waist/hip ratio | 0.98 (0.95-1.02) | 0.98 (0.95-1.02) | 0.639 |
| Medical history |  |  |  |
| Hypertension | 737 (63.9) | 510 (66.0) | 0.342 |
| Hyperlipidemia | 367 (31.8) | 270 (34.9) | 0.153 |
| Diabetes | 345 (29.9) | 264 (34.2) | 0.049 |
| Prior PCI | 238 (20.6) | 159 (20.6) | 0.904 |
| Prior stroke | 127 (11.0) | 80 (10.3) | 0.649 |
| Prior MI | 195 (16.9) | 121 (15.7) | 0.470 |
| Current smoking | 530 (45.9) | 388 (50.2) | 0.066 |
| Current drinking | 377 (32.7) | 260 (33.6) | 0.659 |
| Laboratory data |  |  |  |
| Creatinine (μmol/L) | 72.9 (64.1-82.7) | 75.0 (63.4-85.8) | 0.004 |
| Hs-CRP (mg/L) | 2.01 (0.74-5.90) | 1.95 (0.80-6.53) | 0.673 |
| LVEF, % | 61 (56-65) | 61 (56-65) | 0.860 |
| LDL-C, mmol/L | 2.44 (1.89-3.10) | 2.43 (1.91-3.09) | 0.980 |
| HDL-C, mmol/L | 0.99 (0.86-1.17) | 1.00 (0.86-1.15) | 0.953 |
| TC, mmol/L | 4.12 (3.46-4.93) | 4.12 (3.47-4.89) | 0.760 |
| TG, mmol/L | 1.52 (1.11-2.21) | 1.50 (1.07-2.18) | 0.474 |
| HbA1c (%) | 6.00 (5.60-7.00) | 6.10 (5.65-7.05) | 0.387 |
| Systolic BP (mmHg) | 126 (117-138) | 128 (118-138) | 0.518 |
| Diastolic BP (mmHg) | 75 (70-84) | 76 (70-85) | 0.277 |
| Sleep information |  |  |  |
| AHI (events per hr) | 16.00 (7.90-31.22) | 16.10 (8.10-29.50) | 0.861 |
| ODI (events per hr) | 16.30 (8.60-29.00) | 16.10 (9.20-27.85) | 0.817 |
| Minimum SaO2 (%) | 85 (81-88) | 85 (80-88) | 0.070 |
| Mean SaO2 (%) | 94 (93-95) | 94 (93-95) | 0.225 |
| Time with SaO2 < 90% (%) | 2 (0.4-10) | 3 (0.3-9.7) | 0.918 |
| Epworth Sleepiness Scale | 7.67 ± 4.93 | - | - |
| Diagnosis |  |  | 0.870 |
| STEMI | 253 (21.9) | 177 (22.9) |  |
| NSTEMI | 221 (19.2) | 144 (18.6) |  |
| Unstable angina | 680 (58.9) | 452 (58.5) |  |
| Non-obstructive CAD |  |  |  |
| MINOCA | 25/474 (5.3) | 12/321 (3.7) | 0.322 |
| INOCA | 59/680 (8.7) | 22/452 (4.9) | 0.015 |
| Procedures |  |  |  |
| Coronary angiography | 1126 (97.6) | 752 (97.3) | 0.603 |
| PCI | 618 (53.6) | 433 (56.0) | 0.815 |
| PTCA | 100 (8.7) | 59 (7.6) |  |
| CABG | 86 (7.5) | 45 (5.8) |  |
| Multivessel disease | 723 (62.7) | 523 (69.5) | 0.021 |
| Number of stents | 1 (0-1) | 1 (0-1) | 0.359 |
| Medications on discharge |  |  |  |
| Aspirin | 1121 (97.1) | 756 (97.8) | 0.371 |
| P2Y12 inhibitor | 1057 (91.6) | 711 (92.0) | 0.763 |
| ACEI or ARB | 700 (60.7) | 495 (64.0) | 0.134 |
| CCB | 233 (20.2) | 170 (22.0) | 0.341 |
| β-blockers | 893 (77.4) | 595 (77.0) | 0.833 |
| Statins | 1135 (98.4) | 762 (98.6) | 0.698 |

Abbreviations: ACEI, angiotensin-converting enzymes inhibitor; AHI, apnea-hypopnea index; ARB, angiotensin receptor blocker; BMI, body mass index; BP, blood pressure; CABG, coronary artery bypass grafting; CCB, calcium channel blockers; EDS, Excessive daytime sleepiness; ESS, Epworth Sleepiness Scale; HDL-C, high-density lipoprotein cholesterol; Hs-CRP, high-sensitivity C-reactive protein; INOCA, ischemia with non-obstructive coronary artery disease [defined as angina with non-obstructive CAD (<50% diameter stenosis)]; LVEF, left ventricular ejection fractions; LDL-C, low-density lipoprotein cholesterol; MINOCA, myocardial infarction with non-obstructive coronary artery disease [defined as MI with non-obstructive CAD (<50% diameter stenosis)]; NSTEMI, non-ST-segment elevation myocardial infarction; ODI, oxygen desaturation index; OSA, obstructive sleep apnea; PCI, percutaneous coronary intervention; PTCA, percutaneous transluminal coronary angioplasty; SaO2, arterial oxygen saturation; STEMI, ST-segment-elevation myocardial infarction; TC, total cholesterol; TG, triglyceride.

Data are presented as mean ± standard deviation, median (first quartile, third quartile), n (%).


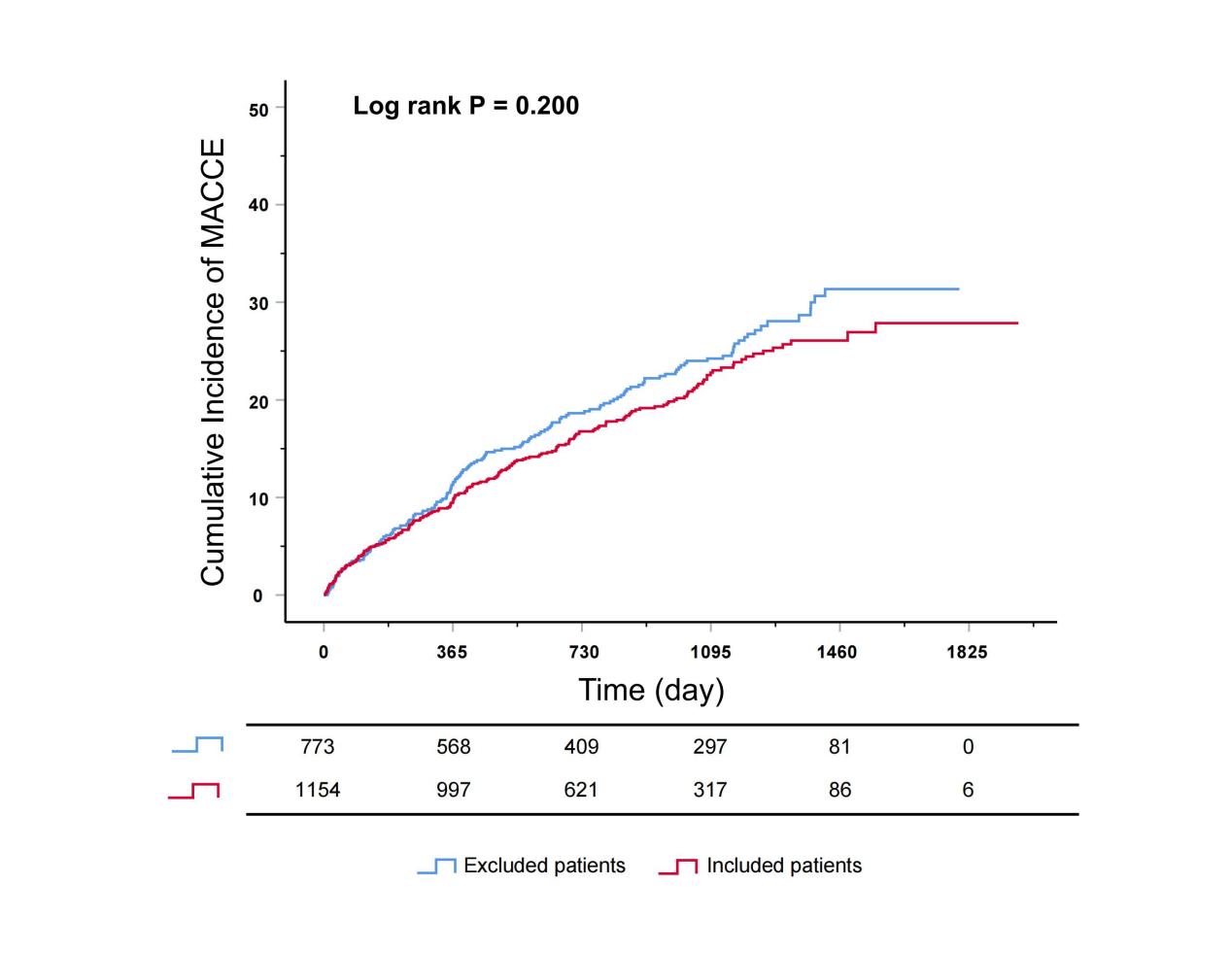


Supplementary Fig. 2. Kaplan-Meier curves for included patients versus excluded patients. Abbreviations: ACS, acute coronary syndrome; MACCE, major adverse cardiovascular and cerebrovascular event; ESS, epworth sleepiness scale; OSA, obstructive sleep apnea.
