# Supplementary figures and images for: Association of Obstructive Sleep Apnea With Cardiovascular Events in Acute Coronary Syndrome Patients With or Without Excessive Daytime Sleepiness: A Prospective Cohort Study
Source: Rev Cardiovasc Med. 2025 Jul 28;26(7):33439. doi: 10.31083/RCM33439 (PMC12326451; doi:10.31083/RCM33439)

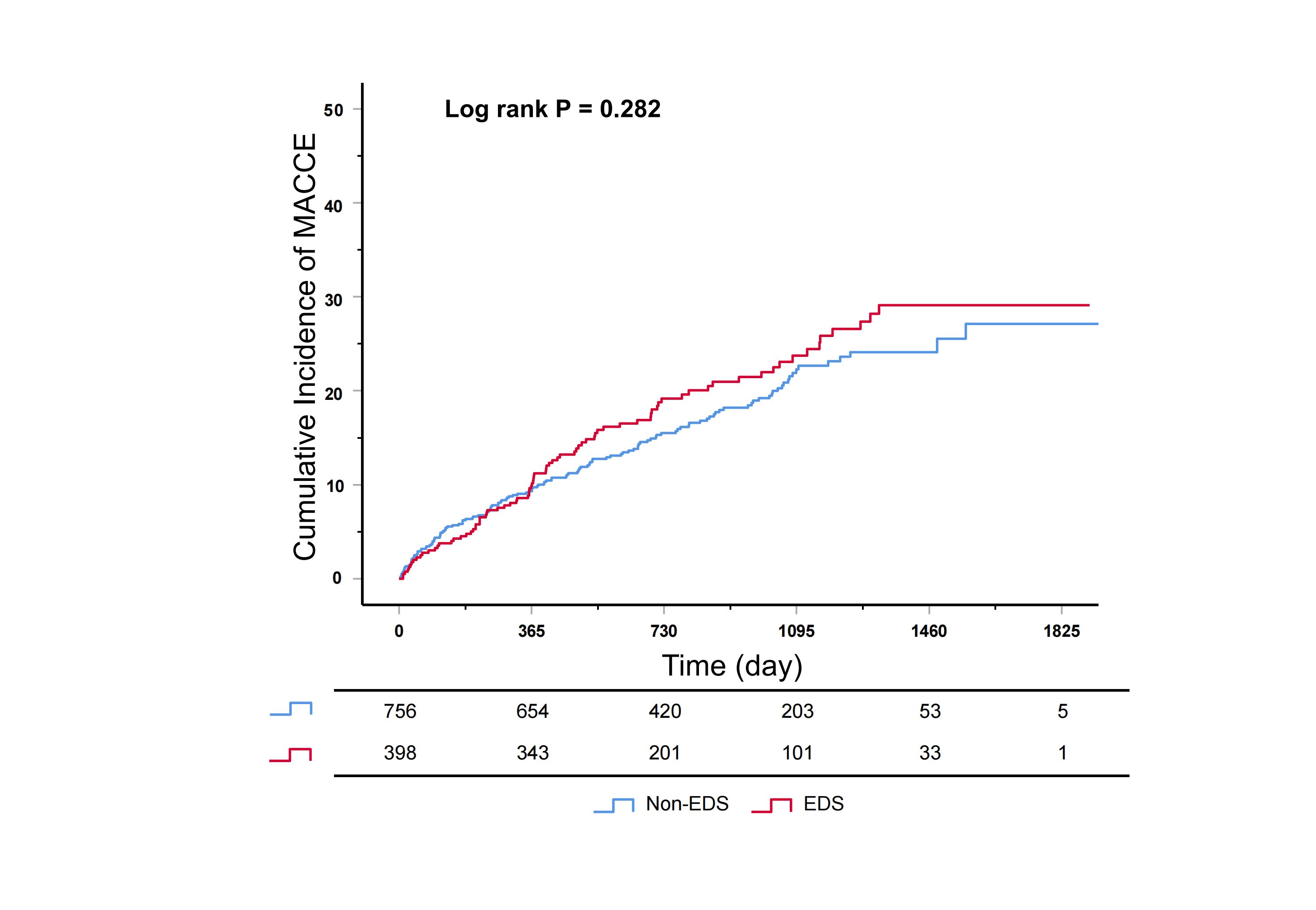

Supplement: Supplementary file 1 [file 2153-8174-26-7-33439-s1.zip › Supplementary Fig. 1.tif]

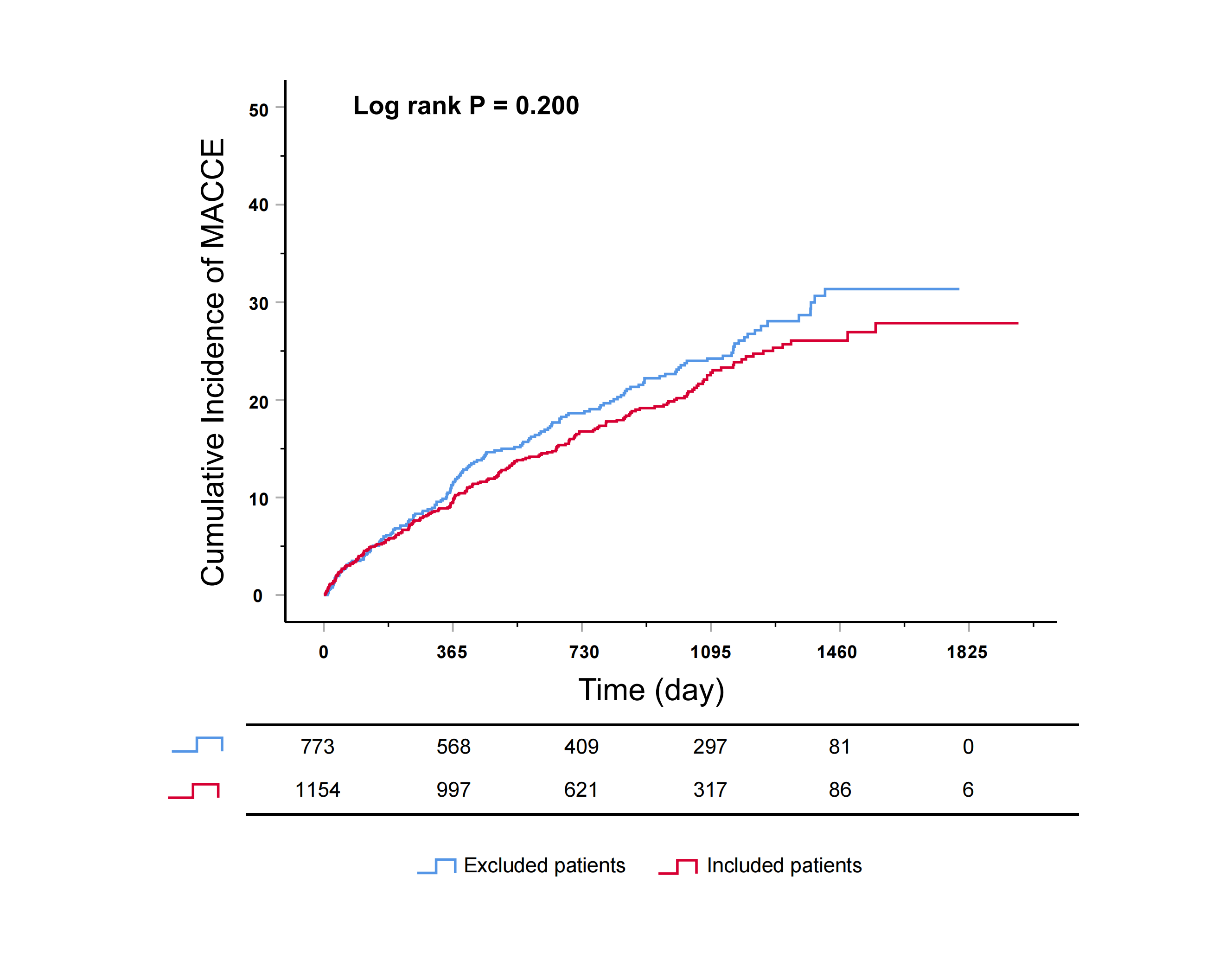

Supplement: Supplementary file 1 [file 2153-8174-26-7-33439-s1.zip › Supplementary Fig. 2.tif]
